# Carcinome épidermoïde compliquant la maladie de Verneuil

**DOI:** 10.11604/pamj.2017.27.139.3020

**Published:** 2017-06-28

**Authors:** Sanaa Lemtibbet, Badreddine Hassam

**Affiliations:** 1 Service de Dermatologie, CHU Ibn Sina, Rabat, Maroc

**Keywords:** Maladie de Verneuil, dégénérescence maligne, carcinome, Verneuil's disease, squamous cell, carcinoma

## Image en médecine

La maladie de Verneuil (MV) est une affection chronique suppurative fistulisante et d'évolution cicatricielle des follicules pilo-sébacés des régions cutanées où sont présentes des glandes apocrines. La dégénérescence maligne ou la survenue de carcinome épidermoïde au cours de la Maladie de Verneuil est une complication rare à long terme. La fréquence est estimée à 1,5-3%. Une prédominance masculine est notée. La région périnéo-fessière est le plus à risque. Un délai de 23 ans en moyenne est nécessaire entre le début de la Maladie de Verneuil et la dégénérescence. L'histologie montre le plus souvent un carcinome épidermoide bien différentié. C'est une tumeur de croissance habituellement rapide, souvent invasive, à risque métastatique non négligeable (40% cas), avec risque de décès en quelques mois. Nous rapportons le cas d'un patient âgé de 42 ans présentant une maladie de Verneuil de localisation périnéo-fessière évoluant depuis 20 ans et traitée irrégulièrement par des antiseptiques et des cyclines per os. Le patient rapportait l'apparition il y a un an sur un tissu cicatriciel inter-fessier d'une lésion verruqueuse d'évolution progressivement végétante. La biopsie cutanée a révélé un carcinome épidermoide bien différencié et infiltrant. Le bilan d'extension était négatif. Le traitement consistait en l'exérèse de la lésion. L'évolution était favorable avec un recul de 3 mois.

**Figure 1:**
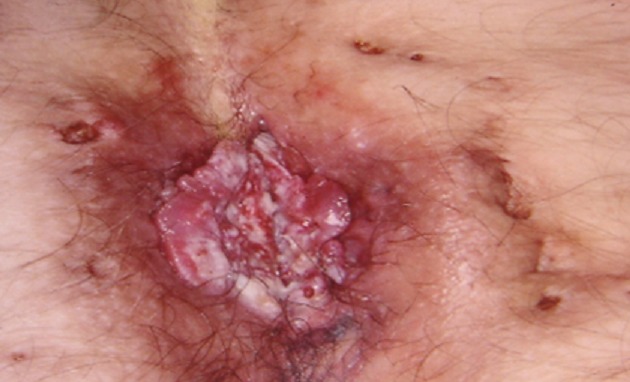
Processus verruqueux et bourgeonnant au centre correspondant à un carcinome épidermoide avec présence en péripherie d’hidrosadénites caractéristiques de la maladie de Verneuil

